# Features and Initial Assessment of the Italian Behavioral Risk Factor Surveillance System (PASSI), 2007-2008

**Published:** 2010-12-15

**Authors:** Sandro Baldissera, Stefano Campostrini, Nancy Binkin, Valentina Minardi, Giada Minelli, Gianluigi Ferrante, Stefania Salmaso, the PASSI Coordinating Group

**Affiliations:** National Institute of Public Health, Rome, Italy; Department of Statistics, Ca' Foscari University, San Giobbe, Cannaregio; National Institute of Public Health, Rome, Italy; National Institute of Public Health, Rome, Italy; National Institute of Public Health, Rome, Italy; National Institute of Public Health, Rome, Italy; National Institute of Public Health, Rome, Italy

## Abstract

**Introduction:**

Surveillance systems for health status and behaviors of populations are fundamental for planning, implementing, and monitoring preventive interventions. In 2006, the Italian Ministry of Health provided funding to the National Institute of Public Health to develop an ongoing surveillance system for adult behavioral risk factors. We describe the main features of the system (known as PASSI) and provide a preliminary assessment of its activity.

**Methods:**

PASSI is conducted by participating local health units, which use a common questionnaire and methods. Each month, local health unit staff conduct telephone interviews of a random sample of resident adults aged 18 to 69 years. Data are transmitted to the national coordinating center, where they are cleaned, managed, and made available for local, regional, and national analysis. Training, data analysis, and communications are centrally supervised, and data quality is routinely monitored.

**Results:**

In 2007 and 2008, nearly 60,000 interviews were completed. The demographic characteristics of survey participants closely corresponded to census data in the surveyed areas. The response rate was 82%; the refusal rate was 10% or less. Communications activities have been conducted to disseminate the results and encourage their use.

**Conclusions:**

PASSI is administered by the public health system with limited human and financial resources. In the first 2 years of activity, the data quality was good, and information collected was useful. The organizational model of PASSI may be of interest to countries that are developing surveillance systems as well as those with systems already in place.

## Introduction

Noncommunicable chronic diseases (NCDs) are responsible for most disease burden worldwide and have a high economic impact (1). In Europe, 86% of all deaths and 77% of disability-adjusted life years lost are attributable to NCDs (2). Given the role of lifestyle in the development of NCDs, the planning, implementation, and evaluation of interventions for their prevention and control rely on timely information about the population's health status and behaviors (1), as well as its knowledge and perceptions of healthy habits. The most appropriate means of obtaining this information is through specific surveillance systems, which must conform with international standards (3,4).

In Italy, the most recent National Health and Prevention Plans include multiple interventions for tackling the most important risk factors for NCDs, in accordance with the integrated approach proposed by the World Health Organization (1). Along with other European countries, Italy adheres to a common strategy for NCD control (Gaining Health) (2) and in 2007 adopted a program called Guadagnare Salute (5).

Italy's National Statistics Institute performs periodic national surveys on health determinants and the adherence to available preventive measures (6). However, the periodicity, timeliness, and local representativeness of these data are often insufficient for planning public health actions. For these reasons, in 2006 the Italian Ministry of Health commissioned the National Centre for Epidemiology, Surveillance and Health Promotion (Centro Nazionale Epidemiologia, Sorveglianza e Promozione Della Salute, or CNESPS) of the National Institute of Public Health to develop a system for the ongoing surveillance of major behavioral risk factors and preventive measures for NCDs. The system, Progress by Local Health Units Towards a Healthier Italy (Progressi Delle Aziende Sanitarie per la Salute in Italia, or PASSI), is based on ongoing nationwide collection of data using a standardized questionnaire. We describe the system and present the results of an initial assessment of its first 2 years of activity. We evaluate population representativeness, quality of the data collection process, acceptability, costs, and sustainability of the system. Our methods are similar to those adopted in previous assessments of the initial phase of surveillance systems in the United States (7-9).

## Methods

At the request of the Ministry of Health, in 2005 and 2006, pilot studies were conducted in most of Italy's 21 regions to assess the feasibility of performing surveillance and providing local and regional data for public health interventions; the pilot studies were coordinated by CNESPS. A provisional protocol was developed by participants in the Field Epidemiology Training Program, in cooperation with regional public health professionals, based on the Behavioral Risk Factor Surveillance System of the US Centers for Disease Control and Prevention (3).

In 2006, a national committee was established to develop the definitive protocol for PASSI. The committee consisted of staff from CNESPS, graduates of the Field Epidemiology Training Program who had performed the pilot studies, and outside experts. The protocol, which has been published elsewhere (10), was approved by the ethics committee of the National Institute of Public Health.

In Italy, health care is provided by the regions, which have considerable economic and regulatory autonomy within the framework of the National Health System. The number of local health units (LHUs) per region varies between 1 and 22, with catchment populations ranging from 40,000 to more than 1 million. Because most public health interventions are planned and evaluated locally, we wanted to maintain the level of "information production" as close as possible to the level of "information use" by involving regions and LHUs directly in the surveillance process.

In PASSI, LHU personnel receive training and support from regional coordinating groups. The national coordinating group, based at CNESPS, provides leadership on technical issues, supports the regional coordinating groups, and oversees the functioning of the system.

Initially, the national coordinating group provided training on all aspects of the system to the regional coordinators. They in turn provided initial training on basic procedures to all PASSI supervisors and interviewers at the LHU level (more than 1,000 nationwide).

The questionnaire was based on questionnaires used for similar surveys, in particular the Behavioral Risk Factor Surveillance System, to facilitate the comparison of PASSI results with those from other systems (11,12).

**Box:** Questionnaire Topics, Italian Behavioral Risk Factor Surveillance System (PASSI), 2007-2008

**Box. Questionnaire Topics, Italian Behavioral Risk Factor Surveillance System (PASSI), 2007-2008**		

**Topic**	**Target Population**
**Fixed core modules**	
Self-perceived health and quality of life	All
Smoking habits	All
Alcohol consumption	All
Cervical cancer screening	Women, 25-69 y
Breast cancer screening	Women, 40-69 y
Colorectal cancer screening	Women and men, 50-69 y
Diet and nutritional status	All
Physical activity	All
Mental health	All
Cardiovascular risk factors	All
Influenza vaccination	All
Rubella vaccination	Women, 18-49 y
Prevention of traffic accidents	All
Prevention of domestic injuries	All
Sociodemographic aspects	All
**Regional optional modules (since June 2008)**	
Prevention of traffic accidents (supplement)	All
Hormone replacement therapy	Women, 45-60 y
Child health promotion campaign	All
Abbreviation: PASSI, Progressi Delle Aziende Sanitarie per la Salute in Italia (Progress by Local Health Units Towards a Healthier Italy).	

The questionnaire covers a variety of topics related to health behavior and prevention, all of which are considered priorities in the National Health Plan (www.ministerosalute.it/dettaglio/phPrimoPiano.jsp?id=316) and the National Prevention Plan (www.ccm-network.it/en_National_Prev_Plan) (Box). Particular attention is given to subjective aspects, such as the respondents' perceptions, opinions, knowledge, and attitudes about health behaviors, and whether their doctors provide them with appropriate medical advice. Almost all questions are closed-ended, with multiple-choice answers. Many questions are administered only to specific population subgroups.

The questionnaire has 4 parts:

1. Fixed core component: a standard set of 114 questions, grouped into 15 modules (Box). These questions are asked by all participating LHUs and are meant to remain substantially unchanged for many years.
2. Rotating core: questions on different topics that are asked in alternating years by all participating LHUs.
3. Optional modules: questions that regions elect to use to satisfy specific regional information needs (eg, for health promotion campaigns).
4. Emerging modules: a few questions, administered for brief periods, to gather timely information on important issues of a "late breaking" nature (eg, influenza).

The questionnaire is revised annually so that new questions can be added (or existing ones reworded), based on public health policy and emerging evidence on the issues addressed by the surveillance system. In the first 2 years of data collection (2007-2008), the fixed core had 2 minor revisions, and 3 optional modules were added. In late 2009, an emerging module on the A-H1N1 influenza virus was implemented, and in 2010 a revised version of the questionnaire was introduced that included a rotating module and 2 new optional modules.

In each LHU, a random sample is extracted each month from the enrollment lists of residents aged 18 to 69 years in the catchment area, stratified by sex and age group (18-34 y, 35-49 y, 50-69 y). These lists contain demographic data (eg, birthday, sex), home address, name of their general practitioner (GP), and often a telephone number. The sample is large enough (at least 25 people per month per LHU) so that annual estimates of the main variables can be obtained with acceptable precision at the LHU level, as well as more frequent estimates for some regions and the entire country. Inclusion criteria and other methodologic details are reported in the Appendix.

After the sample is extracted, a letter is sent to the homes of the selected individuals. The letter explains the purpose of the system and informs the recipients that they will be contacted shortly; their GPs are also informed. Telephone numbers, if not recorded on the enrollment list, are obtained from telephone directories, from the GPs, or from the respondents themselves when they call to make an appointment after receiving the letter. Survey administrators attempt to call at least 6 times on different days of the week (including weekends) and at different times of the day; if a person cannot be reached, a substitute of the same sex and from the same age group is randomly extracted.

The questionnaire is administered via telephone interviews conducted by specially trained personnel from the public health departments of the LHUs. After briefly explaining the objectives to the respondent, the interviewer obtains oral informed consent. The interviewer uses computer-assisted telephone interviewing or printed questionnaires and subsequent data entry on a personal computer. All data are self-reported. Because they are anonymous, they cannot be validated at the individual level.

The ongoing surveillance process, with interviews conducted every month, provides the system with flexibility, since items can be modified over time; it also permits evaluation of trends and seasonal variations. For small areas, more precise estimates can be obtained by aggregating data over time.

Data collected on the printed questionnaire are entered on a personal computer. All the records are encrypted and transmitted to a centralized database, where basic data-quality controls are performed. No personal identifiers appear in the database. PASSI interviewers and supervisors have protected access to the server through a web portal (www.passidati.it/), with individual user names and passwords and differentiated access profiles. Uploaded records are accessible to the local coordinators, and input errors can be corrected online.

The quality of the data collection process is automatically monitored using indicators modeled after international standards (13,14) (Appendix). After all interviews collected in a calendar year are uploaded, data are checked and edited at the central level.

To calculate regional and national pool estimates, the data from the different LHUs are aggregated. Because the LHUs differ considerably in terms of population size, and the sizes of the samples also differ substantially, a weight specific for each LHU stratum is added to each record to account for the number of interviews performed in each of the 6 strata of the LHU's sample and the size of the corresponding strata in the LHU's target population. Edited datasets are subsequently made available for downloading to regions and LHUs. CNESPS performs national-level analyses and provides the regions and LHUs with tools for obtaining local results, such as data analysis plans and codes to run statistical programs.

## Results

Data collection began in 2007 in 19 regions, and 20 regions participated in 2008. The number of participating LHUs was 124 (74%) in 2007 and 136 (84%) in 2008. Approximately 85% of Italy's 18- to 69-year-old population was covered in 2008.

The number of interviews performed rose from 22,006 in 2007 to 37,819 in 2008, of which 13% and 22%, respectively, were computer-assisted telephone interviews. The monthly number of interviews performed in each region varied widely (range, 25-500) because of differences in population size and number of LHUs. The median duration of interviews for both years was 20 minutes.

The demographic composition of the PASSI national pool sample in 2007 reflected the distribution by age and sex of the population in the corresponding LHUs, as determined by the National Statistics Institute. In the PASSI sample, the younger strata were slightly underrepresented and the older strata overrepresented; the maximum difference was 1.1 percentage points compared with official demographic figures (Table).

The eligibility rates for 2007 and 2008 were 92% and 91%, respectively, and the response rate for both years was 82%. The refusal rate was 10% for 2007 and 8% for 2008. Partial interviews and break-offs (Appendix) accounted for less than 1% of all registered interviews.

The National Centre for Disease Prevention and Control (Centro Nazionale per la Prevenzione e il Controllo Delle Malattie, or CCM) provided the National Institute of Public Health with €1 million (US $1.27 million), used for the national coordinating activities and for supporting the regions in the first 3 years of the project. The participating regions and LHUs contributed part-time personnel, and some regions provided incentives for interviewers. The cost of an interview at the LHU level was an estimated €20 (US $25.46), including preparatory activities and data entry but excluding the coordinating costs.

Preliminary results were presented at the 5th International Conference on Behavioral Risk Factor Surveillance in 2007, in Rome, organized by the National Institute of Public Health and the CCM. The national results of the first year of surveillance (2007) were presented in 2008 during an official workshop in Rome. In September 2009, updated PASSI data were presented at a national conference of the Italian program Guadagnare Salute, sponsored by the Ministry of Health. General annual reports and brief reports on specific health topics have been produced and made available to stakeholders. One peer-reviewed article has been published (15). Most regions and many LHUs have already released their surveillance data and used them for local public health activities. National, regional, and local reports, documents, and other relevant material for PASSI are accessible online (www.epicentro.iss.it/passi/english.asp).

## Discussion

Although we are conducting a more complete evaluation of PASSI, following internationally accepted guidelines (16), and validation studies are being performed to compare the results with those provided by other information sources, this article nonetheless highlights some important characteristics of PASSI. First, the study sample appears to be representative of the target population. Second, acceptability by respondents also appears high, as suggested by low refusal rates and the negligible proportion of incomplete interviews. The response rate compares favorably with rates reported in the most successful international surveys (17,18). Furthermore, data collected by PASSI seem to be of practical use, as demonstrated by the fact that many public health agencies use the data for communication, policy making, and health intervention planning.

Perhaps the most important feature of PASSI is that it is carried out by the public health system at the local level, which seems to have many advantages. In particular the sampled individuals were contacted and interviewed by LHU personnel, which seems to have contributed to the high level of participation; LHUs' lists of residents were readily available for sampling; and direct involvement of LHU personnel in managing the system provided them with the opportunity and motivation to identify and monitor the needs of their populations and their perceptions of the preventive interventions offered.

However, PASSI does have some limitations. In particular, as with any health survey, the reliability and validity of self-reported behavior may be problematic (19). Furthermore, people who are younger than 18 years or older than 69 years are not included in the survey. The age range of 18 to 69 years was chosen because minors cannot provide informed consent to be interviewed by telephone and because even mild cognitive impairment, which is present in a sizable proportion of older people (20), can reduce the reliability of the answers. Moreover, many preventive programs monitored by PASSI, such as tumor screening, are recommended for people up to age 70 years. To obtain information on the health status of the elderly, a different approach (known as PASSI d'Argento) is being piloted with assistance from the national coordinating group; this approach focuses more on health issues of the elderly and allows for face-to-face and, if necessary, proxy interviews.

A critical aspect of PASSI is sustainability. The system is still considered experimental, without a stable institutional setting. Local surveillance is conducted by public health staff who have other routine responsibilities. This situation generates fatigue, which is exacerbated by insufficient political and financial support in some regions, where public health infrastructures are more fragile. Another challenge is maintaining a sense of usefulness when prevalences for many of the variables change little over time. Geographic comparisons have proved useful in sustaining the momentum of the US system (8,9), and as the number of collected interviews increases over time, subgroup analyses at the local level will be possible, improving targeting of health interventions.

Despite these limitations, many regions and LHUs support the goal of creating an institutional setting for PASSI. Furthermore, in 2009, the CCM renewed funding for PASSI for 2 years so that it could be implemented throughout the entire country. Another substantial contribution to the improvement of PASSI has been its link to the international health promotion and surveillance community (21). Our experience shows that a surveillance system such as PASSI can operate with a reasonable amount of resources, producing useful data and gaining the support of its main stakeholders. We believe that PASSI is an interesting model both for countries intending to develop similar surveillance systems and those in which such systems are already in place.

## Figures and Tables

**Table.** Representativeness of Sample for Italian Behavioral Risk Factor Surveillance System (PASSI), 2007

**Sex and Age Group**	2007 PASSI Pool, %	2006 Population, %^a^
Men, 18-34 y	14.4	15.5
Men, 35-49 y	17.0	17.1
Men, 50-69 y	18.1	17.1
Women, 18-34 y	14.5	15.1
Women, 35-49 y	17.1	17.0
Women, 50-69 y	18.9	18.2

Abbreviation: PASSI, Progressi Delle Aziende Sanitarie per la Salute in Italia (Progress by Local Health Units Towards a Healthier Italy).

a National Statistics Institute data based on estimates for December 31, 2006, from the same local health units covered by PASSI.

## Appendix. Eligibility and Outcome Rates for the Italian Behavioral Risk Factor Surveillance System (PASSI)

In Progress by Local Health Units Towards a Healthier Italy (Progressi Delle Aziende Sanitarie per la Salute in Italia, or PASSI), the target population consists of all 18- to 69-year-olds who reside in the local health unit (LHU) area. The following equations were used to calculate interview outcomes:

Eligibility Rate =

$\frac{\left(I+P)+R+(\mathrm{eNC}•\mathrm{NC}+\frac{1}{2}\mathrm{TNF}\right)}{\left(I+P)+R+\mathrm{RNE}+(\mathrm{NC}+\mathrm{TNF}\right)}$

Response Rate =

$\frac{\left(I+P\right)}{\left(I+P)+R+(\mathrm{eNC}•\mathrm{NC}+\frac{1}{2}\mathrm{TNF}\right)}$

Refusal Rate =

$\frac{R}{\left(I+P)+R+(\mathrm{eNC}•\mathrm{NC}+\frac{1}{2}\mathrm{TNF}\right)}$

The survey (eligible) population consists of residents aged 18 to 69 years enrolled on the LHU lists who have a telephone number available and can be interviewed by telephone.

The ineligible units in the LHU lists may be either people who no longer belong to the target population (eg, who moved away or died; in 2007 they constituted 2% of the sample) or people who are in the target population but are not eligible according to the protocol, such as those who do not understand Italian, who cannot participate in the interview (eg, because of serious handicaps), or who are hospitalized or institutionalized (3% in 2007).

People who are reached by the survey and discovered to be not eligible (RNE) are excluded and replaced by people who are randomly selected from the same age and sex stratum.

Sampled people who are not reached by the survey are substituted following the same procedures; their eligibility status is unknown and must be estimated indirectly. They belong to 1 of 2 categories:

1) Those for whom a telephone number was not found (TNF) despite an exhaustive search following the protocol procedures (8% of the sampled population in 2007). It was assumed that half of these people actually had telephones and were thus eligible, given the proportion of Italian families who do not have a landline or a cell phone (3.9%), as estimated by the National Statistics Institute (www.istat.it/salastampa/comunicati/non_calendario/20070724_01/testointegrale.pdf).

2) Non contacts (NC): sampled people who have a telephone number available but who could not be contacted, despite repeated attempts, following the protocol procedures. The proportion of eligible units in the non contacts (eNC) is estimated from the proportion of eligible units in the reached population.

Contacts are people who are reached by the survey and confirmed to be eligible. Contacts who refuse to be interviewed or who break off the interview (<50% of applicable questions answered) are counted as refusals (R). Contacts who agree to be interviewed are counted as responses. Interviews are considered complete (I) if contacts answer at least 80% of the applicable questions and partial (P) if they answer 50% to 79% of the applicable questions.

The eligibility rate is defined as the number of eligible units divided by the total number of sampled units, eligible and ineligible. The response rate is the number of complete and partial interviews divided by the number of all potentially eligible units in the sample; it resembles the American Association for Public Opinion Research (AAPOR) response rate 4 (13). The refusal rate is the number of refusals and break-offs divided by the number of all potentially eligible units in the sample; it resembles the AAPOR refusal rate (13).
